# *In vitro* persistence of clinical methicillin-resistant *Staphylococcus aureus* isolates from bone and joint infections after exposure to daptomycin, telavancin, and vancomycin

**DOI:** 10.1128/spectrum.01576-25

**Published:** 2025-10-23

**Authors:** Aliaa Fouad, Joseph L. Kuti, Christian M. Gill

**Affiliations:** 1 Center for Anti-Infective Research and Development, Hartford Hospital 23893, Hartford, Connecticut, USA; 2 Department of Pharmacy, SSM Health Saint Louis University Hospital 25213, St. Louis, Missouri, USA; Rowan University Cooper Medical School, Camden, New Jersey, USA

**Keywords:** persister cells, MRSA, bone and joint infections

## Abstract

Bacterial persistence is linked to chronic infections caused by methicillin-resistant *Staphylococcus aureus* (MRSA). Understanding the persister profiles of daptomycin, telavancin, and vancomycin is crucial because MRSA bone and joint infections (BJIs) are associated with recurrence and treatment failure. Persister formation was assessed using quantitative *in vitro* assays against six clinical BJI MRSA isolates and a quality control ATCC strain. Assays were performed using a mid-exponential phase of bacteria challenged with steady-state drug concentrations of the three agents. Persisters were quantified as the percent survival quantifiable at 24 h compared with the respective control. Persisters were also quantified after three serial passages of daptomycin pre-exposure. The percent survival identified at 24 h was ≤21.7% with no significant differences between daptomycin, telavancin, and vancomycin in quantitative persister assays without daptomycin pre-exposure. After 3 days of *in vitro* daptomycin pre-exposure, the 24 h percent survival significantly increased compared with no pre-exposure in two isolates for the daptomycin group (100% vs 1% and 4.8% vs 0.2%, respectively). The 24 h percent persister was significantly higher in daptomycin compared with vancomycin and telavancin in four isolates. There was no significant increase in 24 h percent persister for telavancin and vancomycin after daptomycin pre-exposure. Daptomycin, telavancin, and vancomycin resulted in similar bacterial survival at 24 h at clinically achievable concentrations. Daptomycin pre-exposure did not alter persistence for telavancin and vancomycin. Considering differences in persister generation, further clinical studies are justified to help differentiate these anti-MRSA agents in the treatment of MRSA BJIs.

**IMPORTANCE:**

Bacterial persistence has been associated with relapsing infections, including infections caused by MRSA. Chronic infections, such as bone and joint infections, are associated with high rates of recurrence, and persistence may play a role. Although daptomycin, telavancin, and vancomycin are widely used for the treatment of bone and joint infections, their persister potential has not been assessed under the same experimental conditions. The present study sought to assess the *in vitro* persister potential of daptomycin, telavancin, and vancomycin against clinical MRSA from bone and joint infections.

## INTRODUCTION

Methicillin-resistant *Staphylococcus aureus* (MRSA) is a primary pathogen implicated in bone and joint infections, including infections involving prosthetic materials (1–3). MRSA infections are associated with higher rates of recurrence and treatment failure in both native and prosthetic bone and joint infections (4, 5). This leads to an increase in healthcare utilization, accompanied by an increase in both morbidity and mortality rates (6). The role of biofilms in MRSA infections, including prosthetic joints, has been well described (3, 7). In contrast, although MRSA is known to form persister cells (8, 9), the role of bacterial persistence in these infections is not as thoroughly understood and deserves further investigation, particularly in the context of bone and joint infections that are prone to recurrence (10).

Bacterial persisters are different from biofilms, as they are dormant bacterial cells that survive bactericidal concentrations of antimicrobials without becoming resistant and might lead to recalcitrant infection after antibiotic removal (10). Bacterial persistence is a distinct process from the development of resistance; however, persisters may ultimately contribute to the development of resistance (11). Since persister cells may represent another important consideration for the recurrence of bone and joint infections, an assessment of the persister formation potential of different antimicrobial agents used for MRSA bone and joint infections is needed.

Vancomycin and daptomycin represent two of the most common antimicrobials used for MRSA bone and joint infections (2, 3). Previous data have observed poor activity of vancomycin against the stationary phase *S. aureus* (12). Similarly, persisters selected after exposure to ampicillin were minimally affected by high concentrations of vancomycin or daptomycin (12). Telavancin is also a treatment option for bone and joint infections due to its *in vitro* potency against gram-positive pathogens associated with such infections, including MRSA (13), once daily dosing, and positive clinical outcomes for patients treated for these infections (14). However, no persister data are available for this antibiotic to our knowledge. The liability of all three agents to develop persisters warrants systematic investigation. In the current study, an *in vitro* comparison of persister formation among clinical MRSA from bone and joint infections after exposure to daptomycin, telavancin, and vancomycin was performed to further differentiate the utility of these agents.

## RESULTS

### Antimicrobial susceptibility testing

The modal MICs for the MRSA isolates used for the quantitative *in vitro* persister studies are presented in Table 1. All isolates were susceptible to all three antibiotics, as per CLSI breakpoints, and MICs were similar across all clinical isolates collected; thus, the random selection of the six for persister evaluation (15).

**TABLE 1:** Modal MICs and 24 h percent survival for daptomycin, telavancin, and vancomycin to the respective control timepoint against six MRSA isolates^*a*^

Isolate ID	MIC (mg/L)			24 h % survival			24 h % survival after daptomycin pre-exposure		
	DAP	TLV	VAN	DAP	TLV	VAN	DAP	TLV	VAN
STA 534	1	0.125	1	1.0 ± 0.8	0.0 ± 0.0	0.2 ± 0.3	100^*b*^**^,^**^*c*^ ± 0.8	0.3 ± 0.4	0.1 ± 0.1
STA 536	1	0.063	1	11.6 ± 12.9	0.3 ± 0.4	0.5 ± 1.0	27.3^*b*^ ±7.2	0.84 ± 0.6	0.1 ± 0.0
STA 540	1	0.063	1	8.0 ± 2.4	0.1 ± 0.0	0.0 ± 0.0	78.2^*b*^ ±29.1	1.1 ± 1.2	0.0 ± 0.0
STA 548	0.5	0.031	1	1.0 ± 1.3	0.2 ± 0.3	0.2 ± 0.3	6.3 ± 8.8	0.0 ± 0.0	0.1 ± 0.1
STA 551	0.5	0.031	1	0.2 ± 0.4	0.0 ± 0.0	0.0 ± 0.0	4.8^*b*^**^,^**^*c*^ ± 0.5	0.0 ± 0.0	0.0 ± 0.0
STA 552	0.5	0.063	1	21.7 ± 41.6	0.5 ± 0.9	0.0 ± 0.0	9.2 ± 4.2	0.0 ± 0.0	0.0 ± 0.0

^*a*^ The 24 h percent survival values are the mean ± SD of two or three replicates for each isolate.

^*b*^ Statistically significant compared to the 24 h percent persister for both telavancin and vancomycin.

^*c*^ Statistically significant compared with the respective drug 24 h percent persister after daptomycin exposure. TLV, telavancin; DAP, daptomycin; VAN, vancomycin. STA 534 24 h percent value for daptomycin after daptomycin exposure was capped to 100% (actual value is 282%).

### Quantitative *in vitro* persister assay

The 24 h percent survival after exposure to clinically relevant average drug concentrations of daptomycin, telavancin, or vancomycin compared with its respective drug-free control at each time point is summarized in Table 1. The 24 h percent survival ranged from 0.2%–21.7%, <0.1%–0.5%, and <0.1%–0.5% for daptomycin, telavancin, and vancomycin, respectively. There was no significant difference in the 24 h percent survival between the three agents for the six tested MRSA isolates. The 24 h percent survival was <0.1% in two isolates for telavancin and lower than 2% for all the isolates. For vancomycin, three isolates had <0.1% survival at 24 h, and all the isolates showed less than 2% survival. The 24 h percent survival for daptomycin was higher than 10% for two isolates (STA 536 and STA 552).

**Fig 1:**
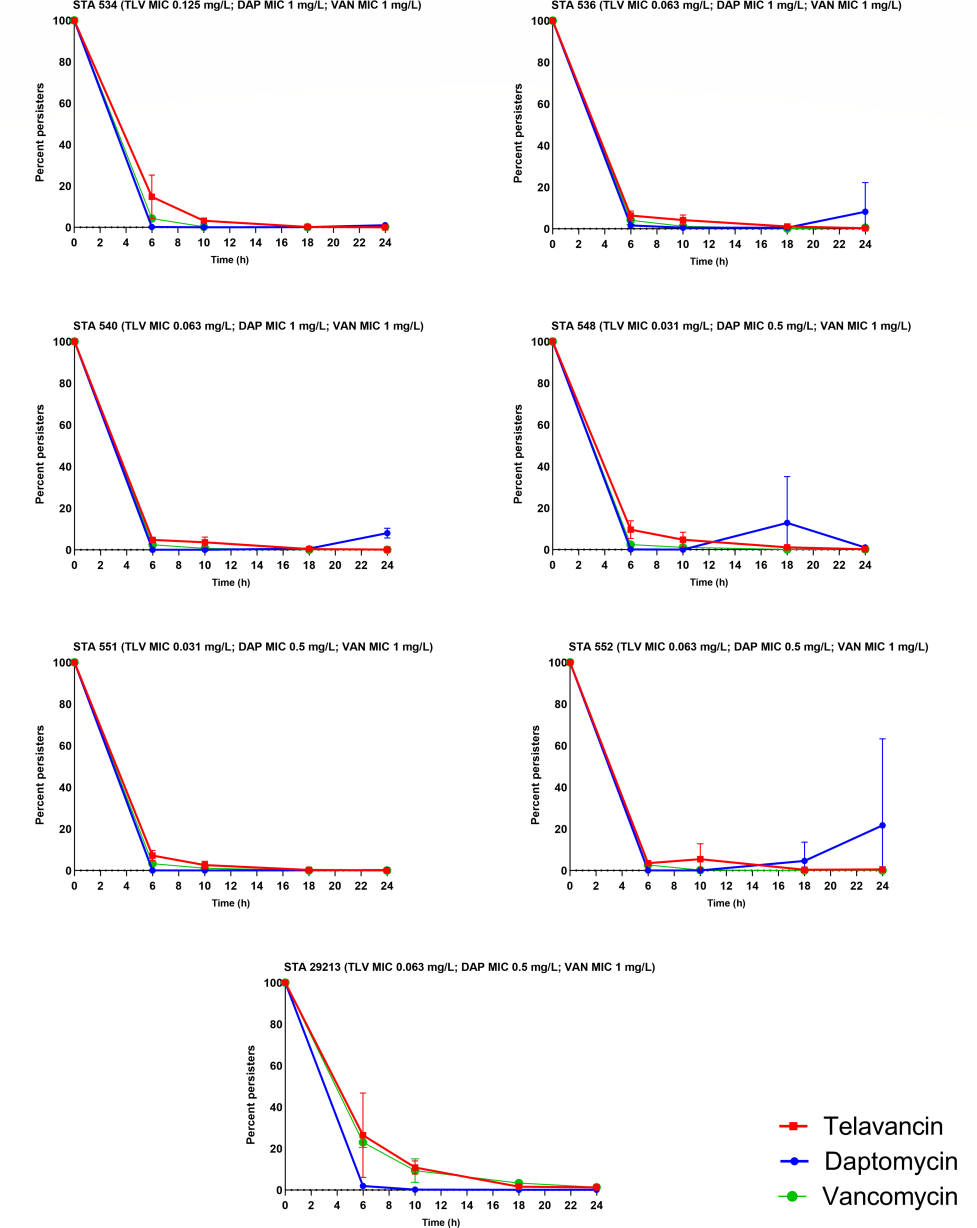
Twenty-four-hour survival curves of six MRSA isolates and one MSSA isolate (STA 29213) at mid-exponential phase treated with steady-state concentrations of telavancin (TLV), daptomycin (DAP), or vancomycin (VAN). Each time point represents the mean values of the two replicates with standard deviations.

In the current study, the ATCC 29213 strain produced low levels of 24 h survival for vancomycin of 1.4%, which is consistent with previous studies, despite slight methodological changes mainly related to the drug concentration used (16). The 24 h percent survival was also 0.1% and 1.3% for daptomycin and telavancin, respectively. The time course of persister development for the six MRSA isolates and the ATCC strain after exposure to daptomycin, telavancin, or vancomycin as a percent of bacterial burden relative to the respective untreated growth control time points is shown in Fig. 1 and portrays the biphasic killing profile of the survival curves consistent with persisters.

Since the 24 h percent survival only represents the activity at a single time point, the log ratio (LR) difference in area under the curve for cfu (AUCFU) was calculated for each antibiotic alone, referenced to the control isolate; hence, the lower LR values indicate a higher magnitude of kill. The LR difference in AUCFU was determined to evaluate the change in bacterial burden between the three agents over the full 24 h experiment. The results of the LR of the AUCFU analyses for the six MRSA isolates are provided in Table 2. The LR values were comparable between the three agents except for STA 534, where the telavancin LR value was significantly higher compared with daptomycin and vancomycin.

**TABLE 2:** Log ratio of AUCFU for each antibiotic (test) compared with the control (reference) against six MRSA isolates^*a*^

Isolate ID	Log difference in AUCFU^*f*^			Log difference in AUCFU^*f*^ after daptomycin pre-exposure		
	DAP	TLV	VAN	DAP	TLV	VAN
STA 534	−2	−1.6^*e*^	−2	−0.1^*b*^^,*d*^	−2.1	−2.1
STA 536	−1.5	−1.4	−1.6	−0.6^*c*^	−1	−1.3
STA 540	−1.5	−1.5	−1.8	−0.5^*b*^^,*d*^	−2.3	−2.4
STA 548	−1.3	−1.4	−1.6	−1.1	−1.7	−1.9
STA 551	−1.6	−1.4	−1.5	−1.5	−2.0	−2.2
STA 552	−1.1	−1.5	−1.9	−1.6	−2.1	−1.8

^*a*^ AUCFU_reference_ is the AUCFU for the control group to assess the activity over a full 24 h. AUCFU_test_ is the AUCFU for each drug.

^*b*^ Statistically significant compared to the LR for both telavancin and vancomycin.

^*c*^ Statistically significant compared to the LR of vancomycin.

^*d*^ Statistically significant compared to the respective drug LR after daptomycin exposure.

^*e*^ Statistically significant compared to the LR of daptomycin and vancomycin.

^*f*^ Log difference is reported as log ratio (LR), which is equal to the number of log_10_CFU (test/reference). AUCFU, area under the curve for CFU. TLV, telavancin; DAP, daptomycin; VAN, vancomycin.

### Quantitative *in vitro* persister assay after daptomycin pre-exposure

Daptomycin pre-exposure trials were performed to determine the appropriate concentration of daptomycin that can be used to achieve a reasonable amount of bacterial load to be transferred over the 3 days of daptomycin exposure. Three different daptomycin concentrations were tested (1× MIC, 2× MIC, and 3× MIC). Passes at 3× the MIC failed to consistently leave sufficient bacteria for subsequent passages. Since both 1× and 2× the MIC were able to complete the three passes, the 2× MIC daptomycin concentration was used to provide the highest test concentration that still provided bacterial load to continue the passes over 72 h.

**Fig 2:**
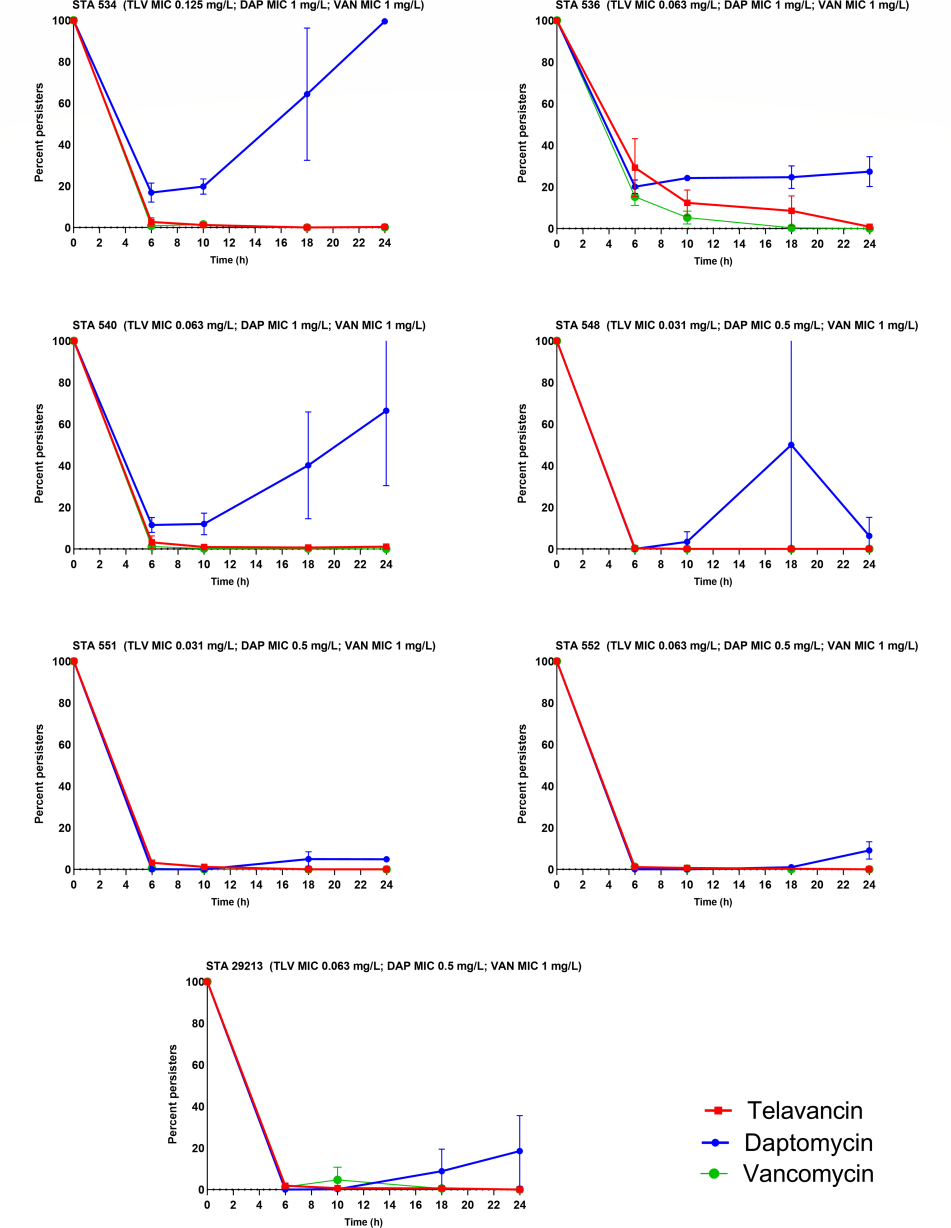
Twenty-four-hour survival curves of six MRSA isolates and one MSSA isolate (STA 29213) at mid-exponential phase after three serial passes of daptomycin (two times MIC) exposure and then treatment with steady-state concentrations of telavancin (TLV), daptomycin (DAP), or vancomycin (VAN). Each time point represents the mean values of the two replicates with standard deviations. The results were capped at 100%.

The time course of persister development for the six MRSA isolates in addition to the ATCC strain after daptomycin pre-exposure to daptomycin, telavancin, or vancomycin as percent of bacterial burden relative to the respective control time point after 3 days of daptomycin exposure is shown in Fig. 2. The 24 h percent survival compared with the respective control time point observed with daptomycin, telavancin, or vancomycin against tested isolates after daptomycin pre-exposure for 3 days is summarized in Table 1.

Exposing the isolates to daptomycin for 3 days at 2× MIC resulted in a significant increase in the 24 h percent survival for daptomycin in two isolates (STA 534 and STA 551) compared with the value of 24 h percent survival without exposure. The 24 h percent survival was significantly higher in daptomycin compared with telavancin and vancomycin in four isolates (STA 534, STA 536, STA 540, and STA 551). Daptomycin pre-exposure did not affect 24 h percent survival for either telavancin or vancomycin compared with the 24 h percent survival without daptomycin pre-exposure. For both agents, the 24 h percent survival remained less than 2% for all seven isolates. The ATCC strain, 24 h percent survival was 18.6%, 0.2%, and 0.03% for daptomycin, telavancin, and vancomycin, respectively.

The results of the LR of the AUCFU analyses are provided in Table 2. LR values after daptomycin pre-exposure were statistically greater in two isolates for the daptomycin group (STA 534 and STA 540). Between the three agents, the LR was statistically higher for daptomycin compared with telavancin and vancomycin in two isolates (STA 534 and STA 540). In addition, for STA 536, the LR value for the daptomycin group was significantly lower than that of the vancomycin group.

## DISCUSSION

Daptomycin, telavancin, and vancomycin are recommended therapies for MRSA in bone and joint infections per IDSA guidance (3). Direct comparisons of persister cell formation after exposure to vancomycin and daptomycin are not available outside of biofilm-based assays (17). Hence, in the current study, an *in vitro* comparison of persister formation between clinical MRSA isolates from bone and joint infections, including prosthetic infection, after exposure to daptomycin, telavancin, and vancomycin was performed.

Methicillin-resistant *Staphylococcus aureus* (MRSA) is a leading cause of persistent human infections and was classified as a high-priority pathogen by the World Health Organization in 2017 (18, 19). Furthermore, MRSA is prevalent in both hospital and community settings, accounting for approximately 60% of all *S. aureus* infections in the United States (20). Bone and joint infections represent difficult-to-treat clinical infections and are known to be associated with significant morbidity and mortality (21). MRSA is a significant pathogen in bone and joint infections, including infections involving prosthetic materials (1–3). Chronic *S. aureus* infections are often confounded with frequent relapses, which result from not only the development of drug resistance to conventional antibiotics but also from the formation of persister bacterial cells (22). Both telavancin and daptomycin have anti-biofilm activity better than that of vancomycin, making them attractive options for the treatment of bone and joint infections, particularly when prosthetic material is involved (23). Bone and joint infections caused by *S. aureus* often require a longer duration of treatment with more than one antimicrobial agent, which is usually associated with adverse drug reactions (3). Bacterial persisters exist in a transient, metabolically inactive state, rendering conventional antibiotics that target essential cellular growth processes ineffective. This leads to high clinical failure rates in antibiotic treatment (22).

Quantitative *in vitro* persister assays using high antibiotic concentrations are one of the fundamental *in vitro* approaches to study persistence (24). In the current study, isolates were allowed to grow in broth for 3 h to reach mid-exponential phase (25). The isolates were then challenged with antibacterial agents at steady-state concentrations (9), and this approach may better reflect clinical use through assessment at physiological exposures. Utilizing the steady-state concentration may provide a well-defined connection to clinical care, as these exposures are achievable in humans. In addition, this practice has also proven effective in other *in vitro* settings, such as assessing synergy testing (26, 27). Six MRSA isolates, in addition to the quality control ATCC strain, were quantified for their persister cell formation using quantitative *in vitro* persister studies (28). The definition of persistence refers to a subpopulation of bacteria that can withstand bactericidal drug concentrations to which they are otherwise fully susceptible. Indeed, in the experiments that lacked daptomycin pre-exposure, the survival curves for all the assessed isolates displayed the bi-phasic kill pattern that is characteristic of persister cells (Fig. 1) (28). The quality control strain ATCC STA 29213 was added to the *in vitro* persister studies for comparison with external data (16). In the current study, the ATCC strain exhibited low levels of persisters in the vancomycin group, consistent with previous research, despite minor methodological changes mainly concerning the drug concentration used (16). There was also no significant difference in survival cell between the three therapies for either the ATCC strain or any of the tested MRSA isolates. It should be noted that two isolates exhibited numerically higher 24 h percent persisters for daptomycin (STA 536 and STA 552), although no statistical significance was observed due to the large variability among daptomycin replicates. This variability was supported by the high values of the standard deviation. Specifically, STA 536 showed a mean ± SD of 11.6 ± 12.9, whereas STA 552 exhibited a 24 h percent persisters of 21.7 ± 41.6. Since there is no standard cutoff that constitutes high-level persistence, quantitative statistical testing was conducted to evaluate differences among the tested agents under the same experimental conditions (24). This also allowed for inference among differences in persisters across the same agent before and after antimicrobial exposure.

Daptomycin and vancomycin are commonly used to treat MRSA infections in bone and joint infections (29), making it crucial to study the effect of pre-exposure to these agents on the production of persister cells since re-treatment may be needed clinically. Similarly, ampicillin pre-treated *S. aureus* isolates were found to cause bacterial persisters, which resulted in blunted antimicrobial activity when exposed to vancomycin and daptomycin (12). Since daptomycin and vancomycin are used so widely, determining activity after exposure may offer translational insights into the activity-specific agents after initial treatment with another. Vancomycin pre-exposure and the activity of telavancin after that were previously reported by Thabit *et al*. The authors found that telavancin was comparable with vancomycin and that telavancin activity was not affected by prior vancomycin exposure (30). On the other hand, the activity of telavancin or vancomycin after exposure to daptomycin is less well described (31). Along with the increased clinical use of daptomycin, assessing persister formation after daptomycin pre-exposure may be of clinical interest (32). Consequently, another set of quantitative *in vitro* persister studies was done in the current study after 3 days of daptomycin pre-exposure to provide a translational assessment of whether telavancin and vancomycin activity is compromised by daptomycin pre-exposure. Treating the isolates with two times the daptomycin MIC for three consecutive serial passes resulted in a significant increase in the percent survival for daptomycin in two MRSA isolates (STA 534 and STA 551) compared with those without pre-exposure. This finding is not surprising, since the isolates were exposed to daptomycin; however, this does provide insights into how changes occurred in our model after the daptomycin pre-exposure. The 24 h percent survival was significantly higher in daptomycin-treated groups compared with vancomycin- and telavancin-treated groups for four isolates (STA 534, STA 536, STA 540, and STA 551). Importantly, there was no significant increase in 24 h percent survival for both telavancin and vancomycin after daptomycin pre-exposure. These data suggest that prior daptomycin exposure may not affect the utility of telavancin or vancomycin.

The percentage of persisters was calculated as the average of the two or three replicates at 24 h post-exposure relative to the untreated control. However, since the percentage of persisters only focuses on a single time point, the AUCFU was calculated to evaluate the entire 24 h period. For *in vitro* studies without daptomycin pre-exposure, the LR values among all isolates for the three agents were not significantly different, suggesting a similar rate and extent of kill across the entire 24 h experiment for each agent. Only one exception was observed where STA 534 resulted in a higher LR for telavancin compared with daptomycin or vancomycin, although 24 h percent survival was similar. This may be due to the relatively low values of the persister cells up to 18 h; hence, collectively, the AUCs were not different between the three tested agents. Considering the experiments after daptomycin pre-exposure, the LR values were significantly higher in two isolates (STA 534 and STA 540) for the daptomycin group relative to telavancin and vancomycin. We did not distinguish between the persister cells and the potentially resistant subpopulation because post-exposure MICs were not conducted, which remains a limitation of the present analysis. This was notable in the cases of STA 534 and STA 540 after daptomycin pre-exposure, when re-exposed to daptomycin, where significant re-growth was observed after initial bacterial reductions at 10 h. However, this may align with the theory suggesting that persistence and resistance are related and can lead to one another (11). The remaining isolates followed the characteristic bi-phasic curves consistent with persisters. When comparing telavancin and vancomycin groups with their respective unexposed experiment, no isolates resulted in significantly different LR, further suggesting the daptomycin pre-exposure did not negatively influence telavancin or vancomycin activity. Due to the relapsing nature of some MRSA bone and joint infections, these data suggest that after daptomycin exposure, telavancin and vancomycin therapy remain valuable treatment options. Clinical correlation of these findings for recurrent bone and joint infections is warranted.

It is essential to distinguish between the mechanisms of action of different drug categories. Glycopeptides, such as vancomycin, inhibit bacterial cell wall synthesis by binding to the D-alanyl-D-alanine terminus of peptidoglycan precursors. This binding prevents the precursors from being incorporated into the growing cell wall, effectively blocking the formation of the vital peptidoglycan layer. In contrast, lipopeptides, like daptomycin, primarily disrupt bacterial cell membranes. They bind to the cell membrane, altering its permeability and causing rapid depolarization due to the leakage of ions like potassium. This disruption inhibits critical cellular processes, including protein, DNA, and RNA synthesis, ultimately leading to rapid bacterial cell death (33). Telavancin is a lipoglycopeptide antibiotic that has a dual mechanism of action against gram-positive bacteria. It inhibits bacterial cell wall synthesis by binding to peptidoglycan precursors, similar to vancomycin, while also disrupting the bacterial cell membrane, which leads to membrane depolarization and increased permeability. This dual action contributes to its potent bactericidal activity (34).

Based on the abovementioned daptomycin mechanism of action, it has been shown that growth arrest can lead to tolerance to the antibiotic daptomycin, like what occurs with many other bactericidal antibiotics. However, although growth arrest usually confers antibiotic tolerance quickly and without requiring metabolic activity, daptomycin tolerance develops through a time-dependent process. This process involves active synthesis and accumulation of peptidoglycan and might be connected to persister cell formation and the observations in the present study (35). This can also can be explained by knowing that resistance to daptomycin is affected by various factors. A comprehensive understanding of the complex mechanisms behind daptomycin resistance involves phenotypic changes in both the cell membrane and the cell wall, driven by alterations in metabolic functions and stress response regulatory mechanisms (36, 37).

It is important to acknowledge some limitations in this study. First, the *in vitro* nature of the study does not reflect the clinical situation, where the antibiotic penetration to the bone tissue might differ. With that said, there is a paucity of data correlating antibiotic exposure in bone to clinical outcomes, which may be related to challenges in sampling and measuring drug concentrations in this matrix (38). To enhance the translational value of this work, the free plasma concentration of standard clinical doses of the studied agents was simulated to assess the *in vitro* activity at clinically achievable concentrations. However, the free steady-state average concentration (*f*Css,avg) only represents a single concentration, and future research may benefit from assessing multiple concentrations and ideally the dynamic concentration time profile achieved in humans after conventional doses. Second, no assessments were conducted on telavancin or vancomycin pre-exposure, which may necessitate further studies. Third, the sampling time points chosen for the current study may limit important dynamics in persister formation extending past 24 h, and further studies over longer time points are warranted. Finally, no MRSA isolates with high vancomycin MIC were included, which could affect the observations. However, MRSA with vancomycin MICs > 1 mg/L remains clinically rare, as most MRSA isolates have vancomycin MICs of 1 mg/L or less (39, 40).

To summarize, among clinical MRSA isolates from bone and joint infections, daptomycin, telavancin, and vancomycin resulted in similar percent persisters at 24 h. Pre-exposure with daptomycin did not significantly affect the percentage of persisters at 24 h for either telavancin or vancomycin. In cases of daptomycin failure, both telavancin and vancomycin should be considered, as they are associated with a lower production of *in vitro* persister cells. A notable strength of this study is its use of clinical isolates; however, clinical studies that evaluate outcomes, most notably recurrence, in relation to persister cell formation would be of great interest.

## MATERIALS AND METHODS

### Antimicrobial agents

Daptomycin (Lot: 86190) and vancomycin (Lot: 237326) were obtained as analytical powders from MedChemExpress (Monmouth Junction, NJ, USA). Telavancin powder (Lot: 71522AA006) was provided by Cumberland Pharmaceuticals (Nashville, TN, USA).

### Isolates and antimicrobial susceptibility studies

MRSA isolated from bone and joint infections (including prosthetic joint infections) were acquired from the Bone & Joint Institute at Hartford Hospital (Hartford, CT) and from SSM Health System Microbiology Laboratory (Saint Charles, Missouri) for consideration in the study. Organism identification and methicillin-susceptibility testing were conducted at each laboratory per local standards, but all other experiments were conducted at the Center for Anti-infective Research and Development (Hartford, CT). Antimicrobial susceptibility testing was conducted for daptomycin, telavancin, and vancomycin, using the reference broth microdilution method per CLSI standards (15, 41). Minimum inhibitory concentrations (MICs) were determined in triplicate, and the modal MIC value was reported. ATCC STA 29213, which is a methicillin-susceptible isolate, was used as a quality control strain for antimicrobial testing and was run in parallel with test isolates as per CLSI guidelines (15). Calcium-supplemented broth was used for daptomycin susceptibility testing, whereas for telavancin, 0.002% Tween broth was used per CLSI recommendations (15).

### Quantitative *in vitro* persister assays

Six clinical isolates were randomly selected from the acquired isolates. In addition, the ATCC quality control strain STA 29213 was to be used for the quantitative *in vitro* persister studies, as this strain has also been included in other persister studies with vancomycin (16). *In vitro* persister experiments were conducted in Mueller-Hinton broth for control and vancomycin groups, calcium-supplemented broth for daptomycin, and 0.002% Tween broth for telavancin. Isolates were tested in four groups (untreated growth control, daptomycin, telavancin, and vancomycin) in duplicate experiments. In the current study, we initially performed two replicates for each experiment. However, when we observed significant variability between the two replicates, we added a third replicate for isolate STA 548. Additionally, for three other isolates (STA536, STA551, and STA552), we conducted two extra replicates in addition to the original two runs, resulting in a total of four replicates for those isolates. *In vitro* persister experiments were conducted with a starting bacterial burden of ~10^7^ CFU/mL. Each antimicrobial agent was tested at approximately the steady-state average concentration. Daptomycin was assessed at 3 mg/L to simulate the free C_ss,avg_ for an 8 mg/kg dose (42, 43). Telavancin was tested at a concentration of 3 mg/L to simulate the average free steady-state concentration (*f*C_ss,avg_) after a 750 mg IV every 24 h dose (30, 44). In clinical practice, vancomycin dosing is individualized to target an AUC of 400–600 mg*L/h for invasive MRSA infections. Thus, accounting for a target AUC of 500 and vancomycin plasma protein binding (~50%), vancomycin was tested at 10 mg/L to simulate the *f*C_ss,avg_ (40, 45). Isolates were sub-cultured on 2 consecutive days onto Trypticase agar with 5% sheep blood. A 0.5 McFarland standard was prepared, and from the suspension, 100 µL was added to the broth test tubes. Isolates were incubated at 37°C for 3 h in a shaking water bath and allowed to grow to reach mid-exponential phase before starting to add the drug (9). At 0 h, the three agents were added to the test tubes at steady-state concentration, and then, the tubes were returned to the water bath. The final volume of the broth, inoculum, and drug was adjusted to be 10 mL prior to incubation in a shaking water bath at 37°C. Samples were collected at 0 h (baseline bacterial burden after the 3 h of incubation and prior to the addition of the antibiotics), 6, 10, 18, and 24 h for CFU enumeration as previously described (28). At each sampling time point, a wash step took place before plating to prevent antibiotic carryover as described previously (28). The same wash step was done for the control groups to ensure consistent sample handling. A 500 µL aliquot of each tube was centrifuged for 20 min at 2,500 rpm at 25°C, the supernatant was decanted, and the sample was reconstituted with 500 µL normal saline and was used for serial dilutions and plating. Samples were serially diluted, plated onto blood agar plates with 5% sheep’s blood, and incubated for 18–24 h to quantify the bacterial densities (CFU/mL).

### Daptomycin pre-treatment quantitative persister assays

Daptomycin and vancomycin remain prominent agents used for the long-term treatment of bone and joint infections. Daptomycin use has expanded, as generic availability has made therapy more cost-effective, and a growing body of evidence has supported its use in MRSA bone and joint infections. With expanded use, assessing the impact of daptomycin pre-exposure on persister potential warrants investigation to assess if pre-exposure compromises activity. Hence, in the current study, the six clinical isolates and the ATCC strain were also exposed to daptomycin prior to assessing persistence with daptomycin, telavancin, and vancomycin as another method to assess differences between persistence potential for each agent.

Isolates were sub-cultured on 2 consecutive days onto Trypticase agar with 5% sheep blood. Isolates were suspended in normal saline to a 5 McFarland standard. The bacterial suspension was added to a tube containing calcium-supplemented broth and daptomycin at two times the MIC of each isolate based on daptomycin-exposure trials. The mixture was incubated overnight prior to serial passage in fresh daptomycin-containing mixtures for a total of three passes as previously described (46). After the three passes in daptomycin, the mixture was centrifuged, excess broth decanted off, and suspended in saline at a 0.5 McFarland standard. The 0.5 McFarland Standard was added to tubes containing broth and containing daptomycin, telavancin, or vancomycin as described above. Quantitative persisters assessment was conducted in duplicate for the three agents as outlined above.

Daptomycin pre-exposure trials were performed for all isolates to determine the appropriate concentration of daptomycin that can be used to achieve a reasonable amount of bacterial load to be transferred over the 3 days of daptomycin exposure. Three different daptomycin concentrations were evaluated for each isolate (1× MIC, 2× MIC, and 3× MIC).

### Data analysis

After the quantitative persister assays for each drug treatment group, persisters were quantitated as the percent survival at each time point compared with the respective control time point CFU/mL for that respective isolate-drug combination (47). The primary endpoint was the percent of persisters at 24 h relative to the control growth at the same time point (24 h).

Since the 24 h percent of persisters only represents the activity at a single time point, the log ratio (LR) difference in AUCFU was calculated for each antibiotic alone referenced to the control isolate as LR= Log_10_(AUCFU_test_/AUCFU_reference_), where AUCFU_test_ is the AUCFU for each drug, and AUCFU_reference_ is the AUCFU for the control group as previously described to assess the activity over a full 24 h (41). AUC was calculated for each drug or control using the log-linear trapezoidal rule (47).

A Student’s *t*-test was conducted to compare the 24 h percent survival for each agent in the quantitative persister studies without daptomycin pre-exposure against the 24 h values after daptomycin pre-exposure for each individual agent. One-way analysis of variance (ANOVA) was conducted to compare the means across groups, followed by post-hoc tests using multiple comparison test (Tukey’s HSD) to identify significant differences between individual groups. Similar analyses were conducted to compare the LR for each agent with and without daptomycin exposure, as well as to compare the LR values for the three agents together. All analyses were performed using GraphPad Prism software version 10.0.3 (La Jolla, CA, USA.), with a *P*-value of less than 0.05 considered statistically significant.
